# Highly Compact Circulators in Square-Lattice Photonic Crystal Waveguides

**DOI:** 10.1371/journal.pone.0113508

**Published:** 2014-11-21

**Authors:** Xin Jin, Zhengbiao Ouyang, Qiong Wang, Mi Lin, Guohua Wen, Jingjing Wang

**Affiliations:** THz Technical Research Center, Shenzhen Key Laboratory of Micro-nano Photonic Information Technology, College of Electronic Science and Technology, Shenzhen University, 518060, Shenzhen, China; Washington State University, United States of America

## Abstract

We propose, demonstrate and investigate highly compact circulators with ultra-low insertion loss in square-lattice- square-rod-photonic-crystal waveguides. Only a single magneto- optical square rod is required to be inserted into the cross center of waveguides, making the structure very compact and ultra efficient. The square rods around the center defect rod are replaced by several right-angled-triangle rods, reducing the insertion loss further and promoting the isolations as well. By choosing a linear-dispersion region and considering the mode patterns in the square magneto-optical rod, the operating mechanism of the circulator is analyzed. By applying the finite-element method together with the Nelder-Mead optimization method, an extremely low insertion loss of 0.02 dB for the transmitted wave and ultra high isolation of 46 dB∼48 dB for the isolated port are obtained. The idea presented can be applied to build circulators in different wavebands, e.g., microwave or Tera-Hertz.

## Introduction

With the development of photonic crystal (PhC) theory, PhCs are widely applied for high efficient optical devices [1]–[4]. Nowadays, many studies for designing novel devices are related to the magneto-optical materials for their nonreciprocal property [5]–[10]. Especially, as an essential component in microwave or optical integrated circuits, the wave circulators based on photonic crystal waveguides (PCWs) and magneto-optical materials have attracted much attention for their ability in avoiding unwanted among sub-component reflections and interferences, which would destroy the functions of the designed systems.

Previously, it was thought that PhCs are not suitable for microwave applications for their large size compared with conventional metallic devices. With the development of microwave materials with refractive index higher than 100, microwave circuits made of PhCs can be very compact. For example, for a microwave device built on a PhC using Ca[(Li_1/3_Nb_2/3_)_ 0.95_ Ti_0.05_]O_3-δ_ (whose dielectric constant is 20 [11]) as the background material and ZnO-Nb_2_O_5_ (whose dielectric constant is 101 [12]) as the high dielectric material, the size can be only a few millimeters in width. This makes it practical for applications of photonic crystals in microwave integrated circuits. Furthermore, in many cases, we have to integrate infrared, optical and microwave devices together where the three kinds of signal may intersect with each other, while metallic devices will block infrared and optical signals, so that only photonic crystals are applicable. Therefore, investigation of microwave devices, e.g., circulators, based on photonic crystal is necessary.

Among different circulators, the three-port circulator is a frequently used one. On the other hand, for large scale of integration, minimizing the insertion loss and the size of devices becomes important. By now, several kinds of PCW circulators have been designed based on the resonator coupling theory in PCWs in triangle-lattice PhCs [13]–[18]. One or more magneto-optical resonators and several ferrite rods are set in these circulators, so that it is difficult to miniaturize them further. Moreover, some components may become large in size when they are designed with triangle-lattice PhCs. Thus, circulators based on non-triangle-lattice PhCs, e.g., square lattice PhCs, are necessary.

In this study, highly-compact three-port and four-port circulators with ultra-low insertion loss in square-lattice square-rod PhCs are proposed, demonstrated and analyzed through finite-element method (FEM). Compared to those conventional and formerly mentioned PCW circulators, ours is much more compact and simpler, because only one single magneto-optical rod is needed and no high quality-factor magneto-optical resonators are required in the structure. Applying the scaling property of Maxwell's equations, one can build similar circulators in different wave bands, e.g., Tera-Hertz (THz) and far infrared bands through scaling of refractive index, dimension, and wavelength.

## Method

### Physical model

**Figure 1:**
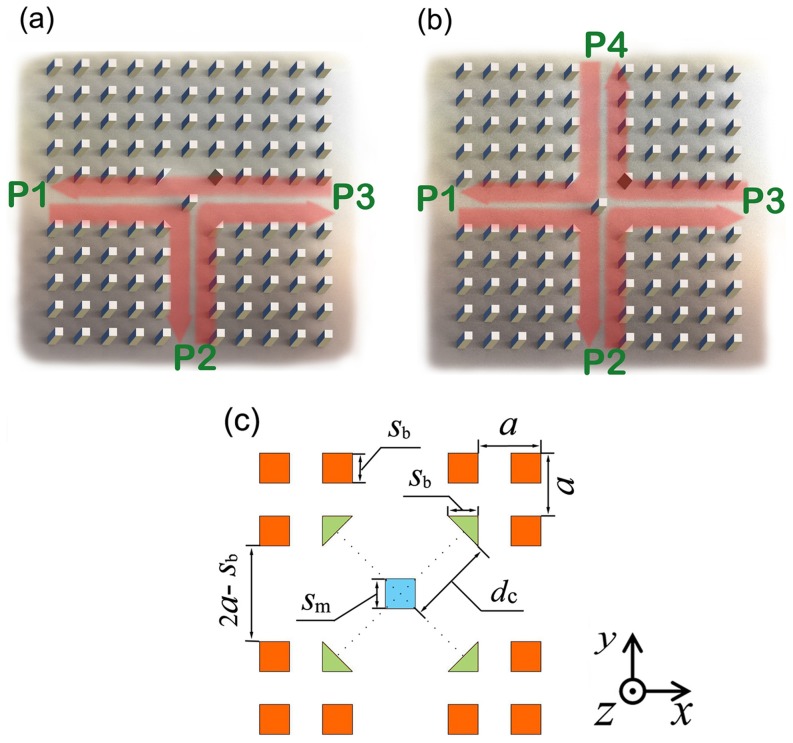
(a) The structure of the ultra-high efficient and highly-compact three-port circulator, (b) is four-port situation. (c) The center region of (b) in which the geometrical parameters are indicated.

Our structures are indicated in Fig. 1. A ferrite defect square rod is set in the center of the PCWs, which are created by taking out a horizontal line and a vertical row of rods respectively from the square-lattice PhC with square silicon rods in air background. Specifically, the four silicon square rods around the PCW center are replaced by four right-angled triangle silicon rods to reduce backward light reflection when the light wave vector rotates 90 degrees. The side length around the right angle of the triangle equals to the side length of the background square rods in the PhC. Moreover, through the four triangle rods, light is also enhanced around the cross center by their function in preventing waves from going to the isolated ports. The ferrite defect square rod is magneto-optical material. It is magnetized when an external D.C. magnetic field is applied. In magnetized state, the ferrite rod can rotate light wave vectors 90 degrees. As depicted in Fig. 1(a), the wave inputted from port 1 (P1), port 2 (P2) or port 3 (P3) should be circulated to P2, P3 or P1, respectively almost without any loss; similarly, in Fig 1(b), the wave inputted from P1, P2, P3 or port 4 (P4) should be circulated to P2, P3, P4 or P1, respectively almost without any loss. It should be noted that, though the waves circulate counterclockwise in all the examples in this paper, the waves will circulate clockwise with a reverse biased D. C. magnetic field. The coordinate system we used in this paper is shown in Fig. 1(c). Furthermore, the geometrical parameters *a*, *s*_b_, *s*_m_, and *d*_c_ are indicated in Fig. 1(c); they are the lattice constant, side width of the square rods (in red) in the PhC, side width of the defect square rod of magneto-optical material (in cyan) and the distance between the cross center and the middle point of the bevel edge of the triangle rods (in green), respectively.

In this paper, we just consider the operation of transverse-electric (TE) mode, which means the electric vector of the wave is transverse to the wave-propagation plane and in parallel to the axis of the silicon square rods. The magneto-optical material has the following relative permeability tensor of 9 elements [19],[20]: [*μ*] = [*μ_ij_*] with *μ*_11_ = *μ*_22_ = *μ*, *μ*_21_ = −*μ*_12_ = *iμp*, *μ*_13_ = *μ*_31_ = *μ*_23_ = *μ*_32_ = 0 and *μ*_33_ = 1, where *i* is the imaginary unit, *p* = *κ*/*μ* is the normalized magnetization frequency, also called as splitting factor. Here *μ* and *κ* are parameters depending on different ferrite materials. Then the Maxwell's equations should take the following form: 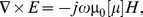 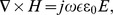 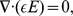 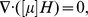 where **E**, **H**, *ω*, *ε*, ε_0_, and μ_0_ are the electric field vector, the magnetic field vector, the operating angle frequency, the relative dielectric constant of the materials, the dielectric constant of free space, and the permeability of free space, respectively. In the following, the proposed circulators are demonstrated and analyzed by solving the Maxwell equations numerically by the commercial FEM software COMSOL RF module. The performance of the circulators for circulating from an input port (P_in_) to an output port (P_out_), while isolating other ports (the isolated port, indicated as P_iso_) from P_in_, can be measured by the insertion loss and isolations: 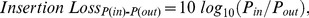
(1)

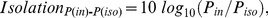
(2)where *P*_in_, *P*_out_ and *P*_iso_ are the power flows at the ports P_in_, P_out_ and P_iso_, respectively. Similarly, as widely accepted, in our study we also neglect the dispersion of silicon and its refractive index is taken as *n* = 3.4.

### Consideration of operating parameters

**Figure 2:**
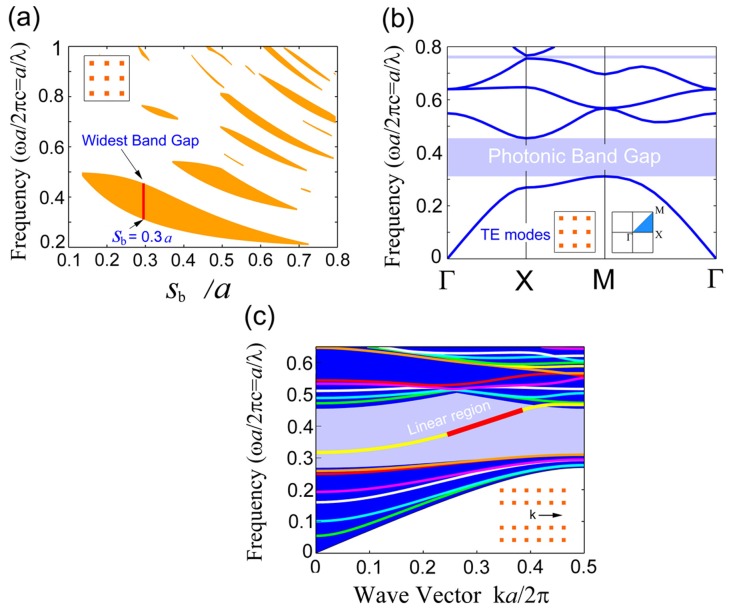
(a) Bandgap map versus radius in the Silicon PhC in which the widest bandgap is drawn in red. (b) The band structure of the Silicon PhC for *r*_b_ = 0.3*a*. (c) The waveguide mode and the projected band structure of the PCW along the wave propagation direction. The yellow colored waveguide mode in the bandgap has a linear dispersion region marked in red.

To confine waves in the PCWs, the frequency of the operating wave should be within the PhC's photonic bandgap. For this, we need to calculate the band structure of the PhC and choose proper size of the square rods in the PhC. The TE bandgap map of the PhC without waveguides is obtained by FEM eigenmode solutions as indicated in Figs. 2(a) and 2(b). Figure 2(a) shows the influence of *s*_b_ on the bandgap width. A maximum bandgap is found for *s*_b_ = 0.3*a*, which corresponds to a bandgap ratio of 36%, as indicated in Fig. 2(a). For this value of *s*_b_, the band structure map is shown in Fig. 2(b). In the following we keep choosing *s*_b_ = 0.3*a* and the operating wavelength within the bandgap.

The waveguide mode in the PhC with a line defect is shown in Fig. 2(c). To minimize the influence of group-velocity dispersion, finding out a range to satisfy d^2^ *ω*/d*k* ^2^ = 0 is necessary [21]. Through calculating the dispersion ratio d*ω*/d*k*, we find an approximately linear dispersion region for the waveguide mode in the following normalized frequency range: *f* = (2πc)^−1^ *ωa* = *a/λ* = 0.3759∼0.4518. In the following, the operating frequency is kept in this range.

### Mode pattern analysis of the defect rod and the operating mechanism of the circulators

**Figure 3:**
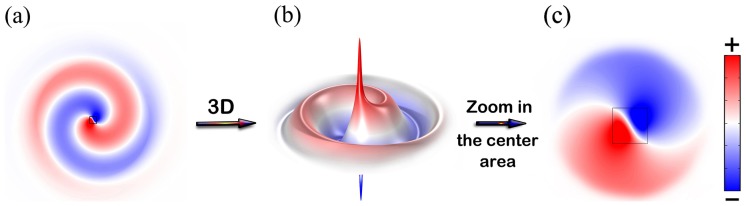
(a) is the electric field distribution *E_z_* in the region with the defect rod when the D.C. magnetic field is applied. (b) is the 3D view of (a). (c) is the zoomed view of the center of (a). Movies of (a) and (b) are available in the Supporting Information list as Movies S1 and S2.

The magneto-optical rod acts as an isotropic material when it is not magnetized, i.e., when there is no D.C. magnetic field. However, it acts as an anisotropic material when it is magnetized. The magneto-optical rod creates a defect within the waveguide. Along with such a defect, there exist resonant modes, but the quality factor for the corresponding resonance modes is generally low so that the operating bandwidth can be wide. The resonant mode patterns, i.e., the electric field distribution, in the defect region can be found by solving Maxwell's equations in isotropic or anisotropic materials using the COMSOL RF module. In obtaining Fig. 3, the splitting factor *p* is taken as 0.77 for the square ferrite with the side width, the relative permittivity and permeability taken as 0.4*a*, ε = 12.9 and *μ*  = 9, respectively. We note that, at present level of material technology, the splitting factor in THz or infrared bands may be much smaller than 0.77. However, with the progress in material science and technology, such a problem may be overcome and we can build circulators accordingly then.

**Figure 4:**
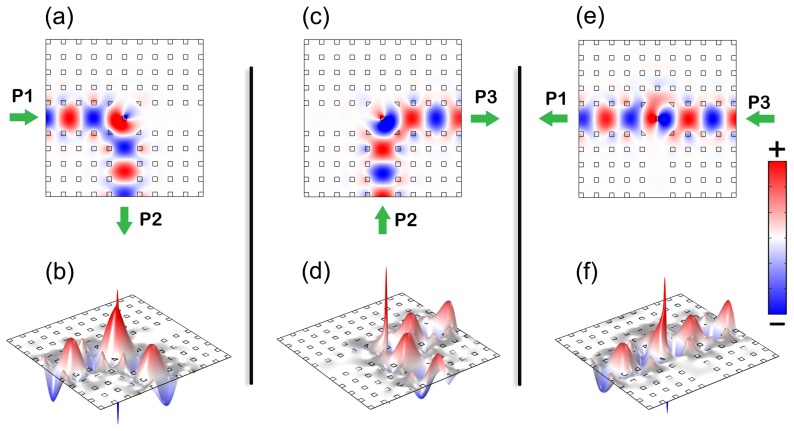
Electric field distribution *E_z_* in the optimized circulator for P1 as input port (a), P2 as input port (c) and P3 as input port (e). Plots (b), (d) and (f) are the 3D version of *E_z_* in (a), (c) and (e), respectively, where the height indicate the value of *E_z_*. The operating parameters are: *s*_m_ = 0.2817*a*, *d*_c_ = 1.2997*a*, *f* = 0.4121, *μ* = 9.6125, and *p* = 0.7792. The animations are available at Movies S3–S8.

The resonant field in and around the magnetized magneto-optical rod is found to be rotating like a mature tropical cyclone of a typhoon, or a spiral-arm galaxy, or the Diagram of the Supreme Ultimate [22]–[23]. To look at the details of the mode center, an enlarged view of it is given in Fig. 3(c) which looks more like the Diagram of the Supreme Ultimate. It is interesting that the Diagram of the Supreme Ultimate can be obtained through Maxwell's equations. Figure 3 (b) is the 3D view of the rotating field in Fig. 3(a), where the height represents the value of the field. One can see from Figs. 3(b) and 4(c) that, there is a positive peak very close to the mode center; also, there is symmetrically a negative peak very close to the mode center, but they two peaks are not at the same point. Moreover, just like the wind speed is zero in the center of a typhoon, the electric field in the center point is always zero because the mode pattern is rotationally anti-symmetric in space. We note that, if we reverse the direction of the D.C. magnetic field in Fig. 3, the rotating direction of the field turns to be counter-clockwise.

The single-direction rotating wave in the magnetized magneto-optical rod explains the mechanism of the circulator. Taking the T-shaped three-port circulator indicated in Fig. 1(a) as an example, when a wave is inputted from P1 (or P2), it will be deflected 90 degrees and most of its power will be transmitted to the adjacent port P2 (or P3) just like a water stream coming from P1 (or P2) and deflected by a stone at the cross position will flow to P2 (or P3). As a result, there is negligible power flowing to port P3 (or P1), which is called as the isolated port. However, the wave inputted from P3 will be deflected 90 degrees twice because the upward path of the wave is blocked and the wave is first deflected upward, then reflected downward to the ferrite region again, and afterwards deflected further to the left side, so that the wave goes to P1 finally. In a similar way, we can understand the operating mechanism of the cross-type four-port circulator indicated in Fig. 1(b).

One may be interested in how the propagation direction of the wave is changed. The reason is that the permeability tensor changes the direction of the magnetic vector of the wave. However, the direction of electric vector of the TE wave is always along the axis of the dielectric rods. As a result, the propagation direction of the TE wave will change accordingly by noting that it should be normal to both the electric and magnetic vectors of the wave. This is why materials with a permeability tensor are necessary for TE-polarization wave circulators. Accordingly, materials with a permittivity tensor is necessary for TM-polarization wave circulators because the magnetic vector has a fixed direction for TM-polarization wave and thus we need materials with a permittivity tensor to change the direction of electric vectors of the wave for changing the propagation direction of the wave in the circulator.

## Results

### T-type circulator

Although the range of operating frequency and the side width of square rods in the PhC are formerly determined, the performance of the circulating are still highly related to the value of the following five parameters: *s*_m_, *d*_c_, *f*, *μ* and *p*. To obtain optimum operating parameters, we used the Nelder-Mead optimization method [24] and define the target function as *G*(*s*_m_, *d*_c_, *f*, *μ*, *p*) = *P*_iso_/*P*_out_ by considering that the value of *P*_iso_ should be as small as possible and *P*_out_ as large as possible for a perfect circulator because the target function is so designed in the optimization program that it has smaller value for better result.

Through optimization with a reasonable computation time, typically about 1 hour on a workstation with 32 cores of 2.4 GHz CPU and 256 G memories, the optimized operating parameters are found to be: *s*_m_ = 0.2817*a*, *d*_c_ = 1.2997*a*, *f* = 0.4121, *μ* = 9.6125 and *p* = 0.7792. The real operating frequency *f*_real_ is 0.3 THz and 30 GHz respectively for *a* = 0.4121 mm and 4.121 mm according to the definition of the normalized frequency *f* = *ωa*/(2πc)  = *f*_real_ *a*/c, where c is the speed of light in vacuum. Under these optimized parameters, the insertion losses in the three cases of operation of the circulator, of which the input ports are respectively P1, P2 and P3, are all as low as 0.02 dB; the corresponding isolations for the three operating cases are 48 dB (P3 as isolated port, P1 as the input port), 46 dB (P1 as isolated port, P2 as the input port) and 46 dB (P2 as isolated port, P3 as the input port), respectively.

To show the effect of the circulator, the field distribution in it is plotted in Fig. 4. Figure 4 demonstrates that the structure works perfectly as a three-port circulator – the wave circulates as desired, the insertion loss is almost zero and there is almost no power flowed to the isolated ports. Figures 4(b), 4(d) and 4(f) are the 3D version of electric fields corresponding to Figs. 4(a), 4(c) and 4(e), respectively. As that in Fig. 3(b), positive and negative peaks of the field are found near the center of the defect rod. Such peaks are originated from the resonance. However, the peaks are not more than three times of the peak field of the input wave, so that the quality factor of the resonance mode is rather low. Therefore, the circulator can provide an operating bandwidth much larger than that of circulators using high-quality-factor magneto-optical resonators [13]–[18]. We noted that in the 2D system for TE mode operation, the rotation of H vector can leads to rotation of k vector according to the relation between **E**, **H** and **k**. Similarly, in the 2D system for TM mode operation, the rotation of E vector can leads to rotation of k vector. This basic principle drives the electromagnetic waves propagating in the system. If the system is not biased by a D.C., we take it as unperturbed. The wave from the left port, for instance, will spread into all the other ports more or less. However if it is biased, the rod in center will perturbed by the field as in Fig. 3. Forced by the spiral-arm galaxy like resonation, the wave transmits into the designated port instead of spreading. Actually, the same spiral motion of waves in the device can be observed both in the center areas of the rods in Fig. 3 and Fig. 4.

**Figure 5:**
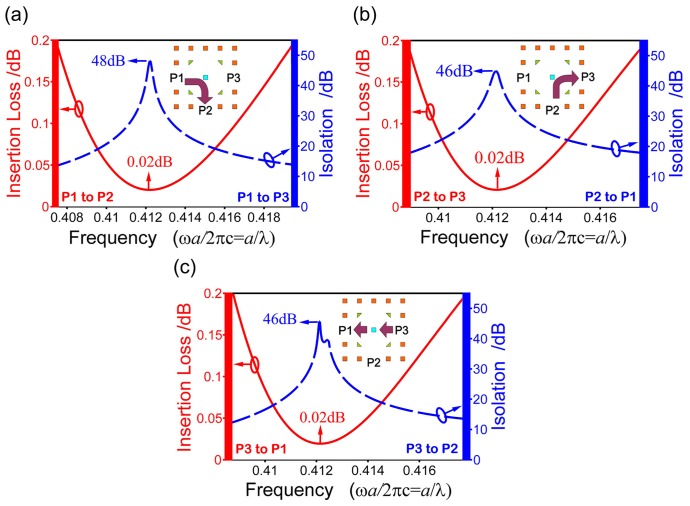
The insertion loss (solid red) and isolation (dash blue) for the first case (P1 as P_in_, P2 as P_out_, P3 as P_iso_) (a), the second case (P2 as P_in_, P3 as P_out_, P1 as P_iso_) (b), and the third case (P3 as P_in_, P1 as P_out_, P2 as P_iso_) (c).

Fixing *s*_m_ = 0.2817*a*, *d*_c_ = 1.2997*a*, *μ* = 9.6125, and *p* = 0.7792 and scanning the frequency *f* around 0.4121, we can get the spectrum response of the optimized circulator, as shown in Fig. 5. From Fig. 5, we can calculate out the 0.2 dB bandwidth, which is defined as the frequency range for the insertion loss less than 0.2 dB, to be (0.4073∼0.4194), (0.4087∼0.4177) and (0.4085∼0.4179), respectively for circulations of P1 to P2, P2 to P3 and P3 to P1. The corresponding relative 0.2 dB bandwidths for the three cases of circulations are 2.94%, 2.18% and 2.28%, respectively. Meanwhile the frequency range for public isolation higher than 15 dB is (0.4088∼0.4182) which is obtained by taking all the three cases into consideration. As a result, taking all the three cases into consideration, we can obtain the overall 0.2 dB bandwidth of (0.4088∼0.4177), which corresponds to a relative 0.2 dB bandwidth of 2.18%. We point out that the insertion loss and isolation can be further improved by simply increasing the lines of rods on the outer sides of the waveguides.

We further point out that the insertion loss will be increased and the isolations decreased obviously if the four triangle rods are replaced by circle rods. This is because the triangle rods are favorable for confining waves around the magneto-optical rods and thus promoting the isolations and decreasing the insertion loss.

### Cross-type circulator

**Figure 6:**
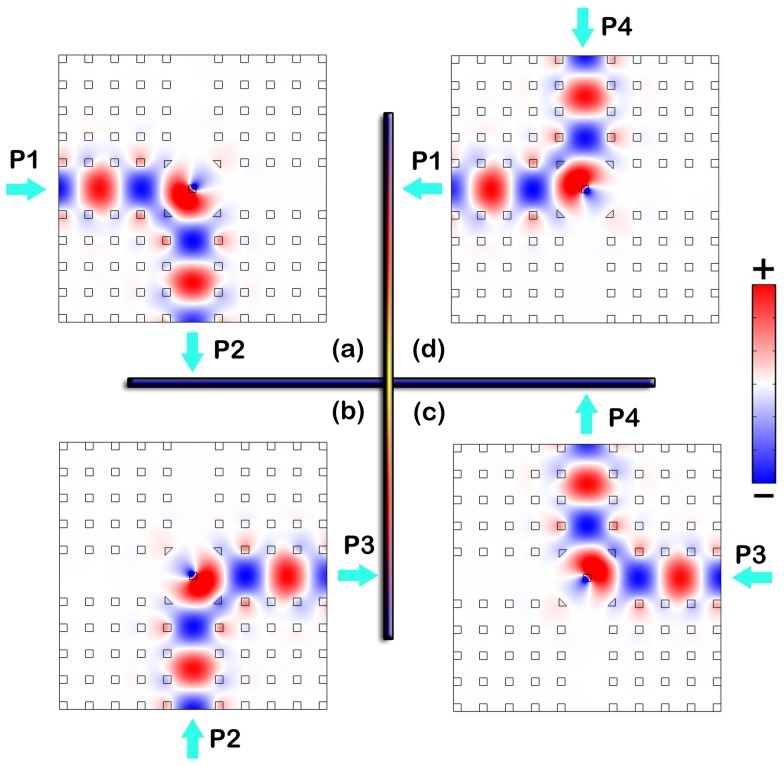
Electric field distribution in the optimized circulator with *s*_m_ = 0.2817*a*, *d*_c_ = 1.2997*a*, *f* = 0.4121, and *p* = 0.7792. The animation for (a) can be observed in Movie S9.

Since the cross-type circulator in our PCW has a 90-degree symmetry about the center, we just need to investigate the circumstance of inputting wave from P1 targeting for P2, i.e. P3 and P4 are isolated ports. Meanwhile, since the central area of the cross-type structure is the same as the one in the T-type one, and the opening of the upper PCW should have negligible influence on the rotation of wave vectors, we can choose the same optimized parameters for the two circulators. Then, disregarding the loss caused by the materials in the PhCs in the system, we can obtain the expected low insertion loss as 0.02 dB for P1-P2 circulation. The corresponding P1–P3 and P1–P4 isolations are obtained to be 46 dB and 48 dB, respectively. To show the effect of the circulator, the field distribution in it is plotted in Fig. 6, which demonstrates that the structure works as a perfect four-port circulator – the wave circulates as desired, there is almost no power flowed to the isolated ports and the insertion loss is almost zero.

**Figure 7:**
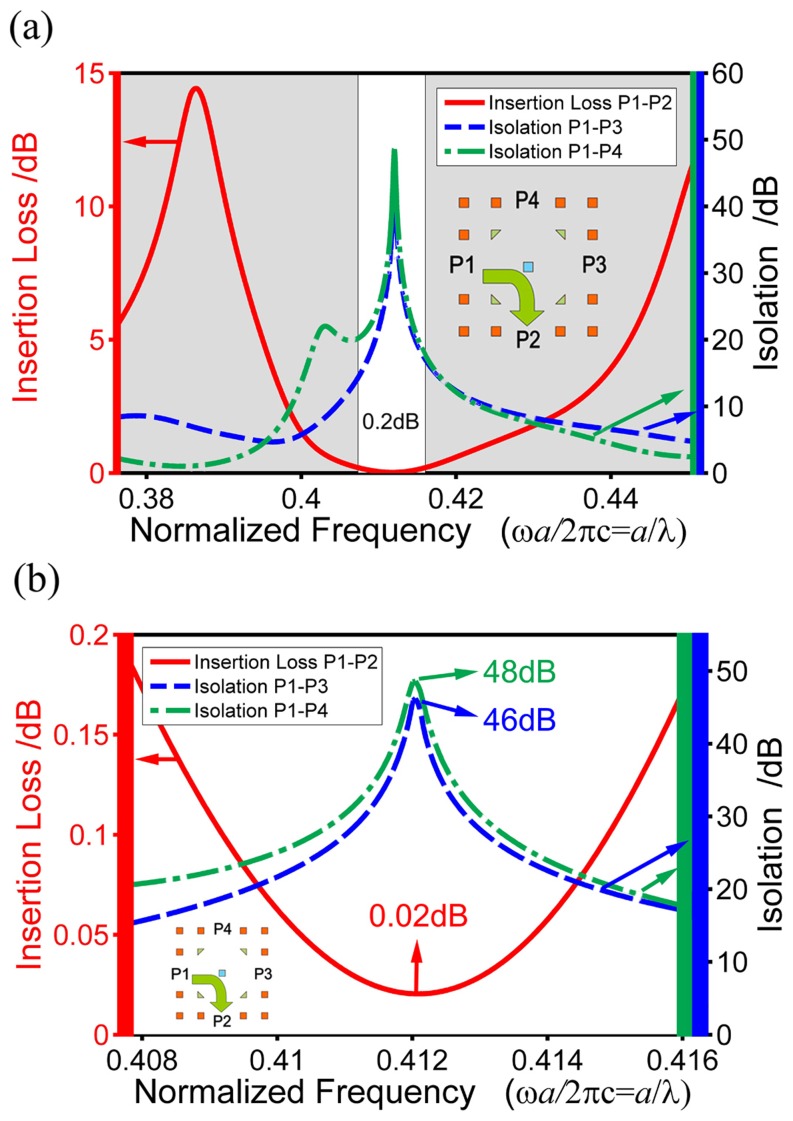
(a) The insertion loss of P1–P2 (solid red) and isolations of P1–P3 (dash blue) and P1–P4 (dot-dash green) in the linear-dispersion region of the waveguide mode range. (b) A zoomed plot for the area with insertion loss less than 0.2 dB in (a).

Fixing *s*_m_ = 0.2817*a*, *d*_c_ = 1.2997*a* and *p* = 0.7792 and scanning the frequency *f* around 0.4121, we can get the spectrum response of the optimized circulator, as shown in Fig. 7. From Fig. 7, we can calculate out the operating bandwidth to be (0.4077∼0.4160) of which the insertion loss is lower than 0.2 dB and the isolations are higher than 15 dB, respectively, with the corresponding relative bandwidth of 2.02%.

## Discussion

In the aforementioned circulator system, only 2D structures are considered for simplicity. For practical device applications, a limitation in the z-direction is required. This kind of limitation in size converts the 2D structures to PhC-slab ones, which are 3D structures. It seemed fine that for PhC slabs with air holes in silicon background, one can use the light cone for confining the wave in z-direction for the wave confining area being dielectric material (dielectric channel) in the PCW. However, to overcome the loss and dispersion that exist in all dielectric materials, one tends to use air channel. Unfortunately, waves cannot be confined in such air channels even if the PhC is of air holes in silicon background, i.e., measures for the third direction confinement of waves is still necessary. Considering further that PhC of silicon rods in air background has much wider photonic bandgap and thus much better confinement of waves than PhC of air holes in silicon background, we choose to use PhC slabs of silicon rods in air background. For the third direction confinement of waves, we propose using substrates and covers of a few periods of 3D PhCs [2], e.g., woodpile, opals and Yablonovite. Specifically, metallic-plate cover and substrate can be used in the microwave region.

**Figure 8:**
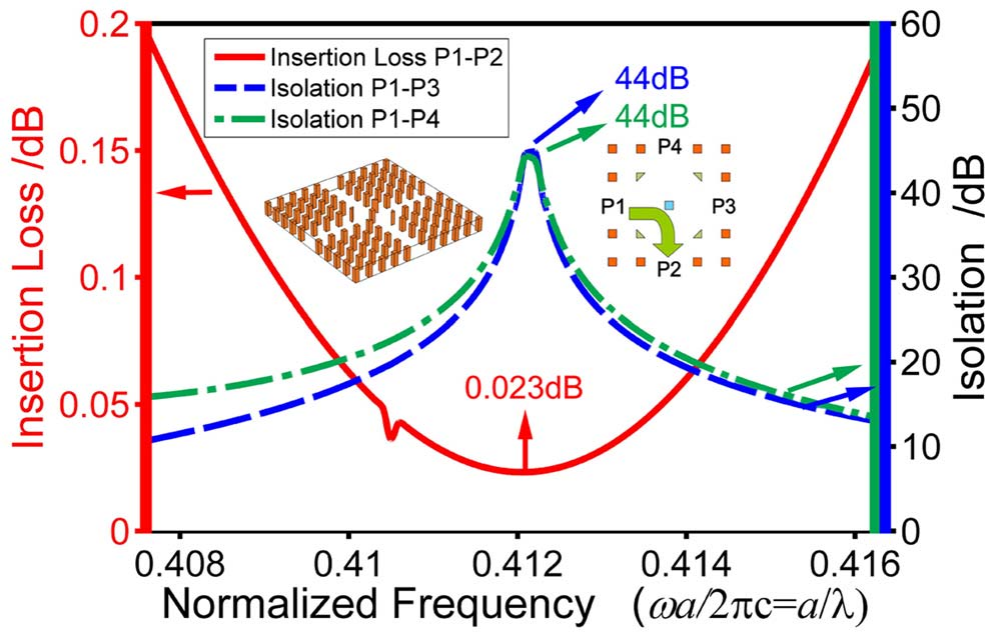
The insertion loss and isolations of the 3D slab circulator with a slab height of *a*. The protocol of color and line-type is same as Fig. 7.

**Figure 9:**
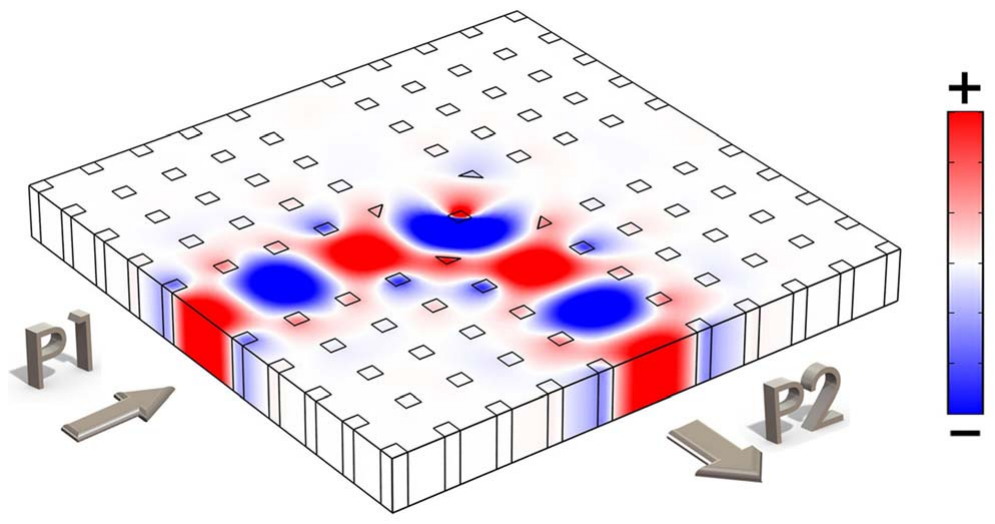
The electric field distribution for circulating wave from P1 to P2 when the normalized frequency is 0.4121 in 3D slab circulator. The animation is available at Movie S10.

We have performed simulation for the 3D slab structure with a cover and a substrate for the cross-type four-port circulator illustrated in Fig 1(b). For the slab with a height of the lattice constant *a*, the simulation result is shown in Fig. 8. The insertion loss is still found to be a very low value of 0.023 dB at the normalized frequency 0.4121. Also, the corresponding P1–P3 and P1–P4 isolations at this frequency are obtained to be both 44 dB. Moreover, the 0.2 dB bandwidth is found to be the same as that for the 2D structure. In Fig. 9, the electric field distribution is displayed, showing that the structure operates as a perfect 3D circulator. We point out that the height of the slab can also be optimized to obtain still better performance [2].

## Conclusions

In conclusion, we have proposed, demonstrated and investigated highly-compact three-port and four-port circulators with ultra-low insertion loss based on the square-lattice square-rod PCWs by FEM. In the circulators, only a single magneto-optical square defect rod is required in the waveguide that makes the structure very compact, the insertion losses very low, and the isolations very high. As another innovative and effective measure, the four square rods around the cross-waveguide center are replaced by four right-angled triangle rods that decreases the insertion losses and increasing the isolations further. At the same time, the linear-dispersion region with high wave speed for the waveguide modes is chosen to minimize the loss and group-velocity dispersion which can cause distortion on the transmitted signal. Furthermore, no high-quality-factor magneto-optical cavities are employed in the circulator, thus a wide operating bandwidth is ensured. The wave in the square defect rod region rotates like a mature tropical cyclone of a typhoon, or a spiral-arm galaxy, or the Diagram of the Supreme Ultimate, which explains the operating mechanism of the circulator. When the loss caused by the materials in the PhCs is omitted, the proposed circulator can have an extremely low insertion loss of 0.02 dB for the transmitted wave and ultra high isolations of 46 dB∼48 dB for the isolated port for all the three cases of operation. The ideas can be applied to build circulators in different wave bands, e.g., THz and infrared bands.

## Supporting Information

Movie S1 **The electric field propagation of Ez in the region with the defect rod.**
(MOV)

Movie S2 **The 3D view of Movie S1.**
(MOV)

Movie S3 **The Ez field propagation of T-type circulator as the input is from P1.**
(MOV)

Movie S4 **The 3D view of Movie S3.**
(MOV)

Movie S5 **The Ez field propagation of T-type circulator as the input is from P2.**
(MOV)

Movie S6 **The 3D view of Movie S5.**
(MOV)

Movie S7 **The Ez field propagation of T-type circulator as the input is from P3.**
(MOV)

Movie S8 **The 3D view of Movie S7.**
(MOV)

Movie S9 **The Ez field propagation of cross-type circulator as the input is from P1.**
(MOV)

Movie S10 **The Ez field propagation of the 3D slab-type cross-type circulator as the input is from P1.**
(MOV)
